# Spatial Navigation Is Impaired in Elderly Patients With Cerebral Small Vessel Disease

**DOI:** 10.3389/fneur.2021.608797

**Published:** 2021-09-08

**Authors:** Hóngyi Zhào, Liyi Chi, Yanhai Zhang, Yonghua Huang, Hongyan Tian

**Affiliations:** ^1^Department of Neurology, Chinese PLA General Hospital, Beijing, China; ^2^Department of Neurology, NO 984 Hospital of PLA, Beijing, China; ^3^Department of Neurology, First Affiliated Hospital of Air Force Military Medical University, Xi'an, China; ^4^Department of Geriatrics, First Affiliated Hospital of Air Force Military Medical University, Xi'an, China; ^5^Department of Cardiology, The First Affiliated Hospital of Xi'an Jiaotong University, Xi'an, China

**Keywords:** aging, cognitive function, spatial navigation, executive function, small vessel disease

## Abstract

Cerebral small vessel disease (SVD) refers to a heterogeneous group of pathological processes that result from damage to the small penetrating vessels in the brain. Spatial navigation, one of the most fundamental behaviors, has lately attracted considerable clinical interest. This study aimed to determine whether spatial navigation performance is impaired in elderly SVD patients. In total, 18 elderly patients with severe SVD, 40 elderly patients with non-severe SVD, and 41 age-matched healthy volunteers were classified according to the Fazekas scale. Spatial navigation was evaluated by Amunet (a computer-based analogy of Morris water maze software), and a mini-mental scale evaluation (MMSE), animal category verbal fluency test (VFT), clock drawing test (CDT), and trail making test (TMT) -B were also applied. Compared to healthy controls, severe SVD, rather than non-severe SVD patients, exhibited significantly worse performance on “allocentric + egocentric” (41.74 ± 29.10 vs. 31.50 ± 16.47 vs. 29.21 ± 19.03; *p* = 0.031). Furthermore, the different abilities of spatial navigation among groups reached a statistical level on allocentric subtests (46.93 ± 31.27 vs. 43.69 ± 23.95 vs. 28.56 ± 16.38; *p* = 0.003), but not on egocentric subtest (56.16 ± 39.85 vs. 56.00 ± 28.81 vs. 43.06 ± 25.07; *p* = 0.105). The linear regression analysis revealed that allocentric navigation deficit was significantly correlated with TMT-B (*p* = 0.000, standardized β = 0.342) and VFT (*p* = 0.016, standardized β = −0.873) performance in elderly SVD patients. These results elucidated that spatial navigation ability could be a manifestation of cognitive deficits in elderly patients with SVD.

## Introduction

Spatial navigation is the process that determines and maintains a trajectory between different points within local environments (1). The success of the navigation process can be influenced by two codependent strategies: egocentric and allocentric navigation strategies (2). The former requires the moving agent to visualize and gauge self-to-object relationships from a body-centered viewpoint, while the latter requires the agent to visualize and map out object-to-object relationships from a disembodied or environment-centered viewpoint (3). Previous functional and structural MRI findings revealed that egocentric navigation might be related to occipital and parietal place area (1), whereas allocentric navigation might be associated more with the hippocampal and prefrontal region (3). Empirically, patients suffering from different types of brain lesions tend to have worse spatial navigation performance. For example, elderly patients with mild cognitive impairment (MCI) and Alzheimer's disease (AD) have a high risk of getting lost behavior (4). Nowadays, several studies have reported that spatial navigation could be modified into a sensitive tool for the preclinical screening of Alzheimer's disease (AD) and dementia (5).

Cerebral small vessel disease (SVD) is a syndrome that involves diseases of the small vessels in the brain, such as white matter hyperintensity lesions, Lacunar Infarctions (LI), etc (6). SVD has received increasing attention in cerebrovascular practice because it is a major cause of vascular dementia and cognitive impairment. The disruption of crucial subcortical connections in the frontal and other lobes, as well as basal ganglia area, following multiple pathophysiological changes such as chronic hypoperfusion, impaired cerebrovascular reactivity, and blood–brain barrier (BBB) leakage, is the core mechanism through which SVD affects cognition (7, 8). As distinguishable from the primary pathological features (cognitive dysfunction) of AD, SVD patients often showed impairments in attention, episodic memory, naming, mental flexibility, and psychomotor speed (6). Although some researchers found that LI was associated with spatial navigation deficits (9), whether the patients with white matter hyperintensities (one of the imaging markers of SVD in MRI) showed spatial navigation abnormalities is still unclear. Besides, we have hypothesized whether the spatial navigation disability in SVD patients was associated with a certain aspect of cognitive decline. In this study, we sought to identify the spatial navigation characteristics of SVD patients by using both egocentric and allocentric strategies and the changes in cognitive function in elderly patients with varying degrees of SVD.

## Methods

### Subjects

From June 1, 2019, to December 31, 2019, a total of 18 and 40 elderly patients with high (severe group) and low (non-severe group) SVD burden, respectively, and 41 age-matched healthy volunteers (control group) were enrolled into this study. All subjects provided written informed consent, and the study protocol was approved by the ethics committee of the Seventh Medical Center of PLA General Hospital. After screening by a 3-Tesla MRI scanner, they were classified based on the Fazekas scale as shown in Supplementary Material 1. The severity of white matter hyperintensities was ranked as follows: grade 0 (no lesion), grade 1 (punctate lesion), grade 2 (early confluent lesion), and grade 3 (confluent lesion). According to the Fazekas scale, the severe group, non-severe group, and control group were defined as grade 2–3, grade 1, and grade 0, respectively. All participants were right-handed, at least 65 years old, and had ordinary visual acuity and other visual capacities. Patients were excluded from the study if they had an intracerebral hemorrhage, major psychiatric disorders, non-vascular dementia, multisystem diseases, taken psychotropic drugs, MRI contraindication, and other causes of leukoencephalopathy (e.g., demyelination, genetic, and immune factors). Basic demographic data, such as age, gender, identity, and educational status, were collected from all participants.

### Neuropsychological Assessment

All subjects were asked to complete a series of neuropsychological tests, including clock drawing test (CDT; reflecting visuospatial function), mini-mental scale evaluation (MMSE; reflecting global cognitive level), trail making test (TMT-B; reflecting cognitive flexibility), and category verbal fluency test (VFT; reflecting semantic memory).

### Spatial Navigation Tests

The Amunet test (a computer-based analogy of Morris water maze software, NeuroScios GmbH, Austria) was used to evaluate the impairment of spatial navigation, which involves both egocentric and allocentric spatial reference systems (9). For the egocentric subtest, subjects were required to navigate a target goal by using the starting position, in the absence of distal orientation cues. For the allocentric subtest, subjects could orientate in a virtual environment using two distal cues, where the starting position was not related to the goal position. In addition, the “allocentric + egocentric” subtest was conducted, which involved the navigation of the goal using both starting position and distal orientation cues (10). Eight trials from each subtest were then used to average the index. Spatial navigational ability was evaluated by measuring the distance between the participants' choice and the correct goal location, which could also be applied as a measure of navigational accuracy.

### Statistical Analysis

The statistical differences in demographic characteristics, CDT, MMSE, TMT-B, VFT, and spatial navigation ability (e.g., “allocentric + egocentric” subtest, egocentric subtest, and allocentric subtest) between the three groups were compared using analysis of variance (ANOVA), with adjustment for age and educational level as covariates. Further comparison of the adjusted means was carried out using a least-squares difference (LSD) test if necessary. A linear regression model (after adjusting for age, sex, and educational level) was used to determine the relationship between spatial navigation impairment and each domain of the cognitive function. All results were presented as mean ± standard deviation, and a *p*-value of less than 0.05 was deemed statistically significant. All statistical tests were conducted using the statistical software package SPSS version 22.0 (IBM Corp., Armonk, New York, USA).

## Results

There were no significant differences among severe SVD, non-severe SVD, and control groups with regards to age, gender, and education level. The MMSE score was decreased in the severe SVD group (24.89 ± 5.70) compared to non-severe SVD and control groups (26.88 ± 3.55 and 27.28 ± 2.04, respectively), but no statistically significant difference was observed (*p* = 0.133). Similarly, the VFT (16.34 ± 6.00 vs. 16.10 ± 7.28 vs. 14.06 ± 6.98) and CDT (9.78 ± 1.39 vs. 9.70 ± 1.58 vs. 8.83 ± 1.66) scores were also not significantly different among the three groups (*p* = 0.462 and *p* = 0.076, respectively). On the contrary, the TMT-B score was significantly lower (89.30 ± 28.98 seconds; *p* = 0.035) in the severe SVD group compared to the non-severe and control groups (74.00 ± 20.47 seconds and 65.70 ± 18.97 seconds). Further details were in Table 1.

**Table 1:** Clinical and demographic characteristics and index of spatial navigation performance of the participants.

	**Severe SVD individuals (*N* = 18)**	**Non-severe SVD (*N* = 40)**	**Healthy (*N* = 41)**	***P* value**
Men, %	55.56%	72.50%	78.05%	0.213
Age, years	71.28 (7.82)	70.75 (7.78)	73.76 (6.77)	0.169
Education, years	12.67 (3.66)	13.05 (3.25)	12.75 (3.55)	0.898
MMSE, score	24.89 (5.71)	26.88 (3.55)	27.24 (4.19)	0.147
VFT, words	14.06 (6.98)	16.10 (7.28)	16.34 (6.01)	0.462
CDT, score	8.83 (1.65)	9.70 (1.59)	9.78 (1.39)	0.076
TMT-B, seconds	65.70 (18.97)	74.00 (20.47)	89.30 (28.98)	0.035^*^^#^
Allocentric+egocentric navigation	41.74 (29.10)	31.50 (16.47)	29.21 (19.03)	0.003^#^^&^
Egocentric navigation	56.16 (39.85)	56.00 (28.82)	43.06 (25.07)	0.105
Allocentric navigation	46.93 (31.27)	43.69 (23.95)	28.56 (16.38)	0.031^#^

*Mean (Standard Deviation)*.

* *P < 0.05 severe SVD relative to non-severe SVD*.

# *P < 0.05 severe SVD relative to healthy individuals*.

& *P < 0.05 non-severe SVD relative to healthy individuals*.

During the allocentric+egocentric subtest, the subjects in severe SVD (41.74 ± 29.10), rather than non-severe SVD (31.50 ± 16.47) groups, performed worse than those in the control group (29.21 ± 19.03; *p* = 0.031). For the egocentric subtest, the performances among the three groups were not significantly different (56.16 ± 39.85 vs. 56.00 ± 28.81 vs. 43.06 ± 25.07; *p* = 0.105). However, for the allocentric subtest, neither the subjects in the severe SVD group (46.93 ± 31.27; *p* = 0.004) nor the subjects in the non-severe SVD group (43.69 ± 23.95; *p* = 0.005) performed as well as healthy individuals (28.56 ± 16.38). These details are shown in Table 1.

Furthermore, the relationship between cognitive task score and spatial navigation ability of elderly SVD patients was determined using linear regression analysis. After adjusting for age, gender, and education level, the average total error in the allocentric subtest was positively associated with TMT-B (*p* = 0.000, standardized β = 0.342), and negatively associated with VFT (*p* = 0.016, standardized β = −0.873). However, the average total errors in “allocentric + egocentric” and egocentric subtests were not significantly associated with the performance indices of neuropsychological tests conducted in this study. More details can be found in Table 2.

**Table 2:** Association between the index of spatial navigation performance and neuropsychological assessment data in aged SVD patients.

	**Allocentric + egocentric navigation**		**Egocentric navigation**		**Allocentric navigation**	
	**Standardized β value**	***P* value**	**Standardized β value**	***P* value**	**Standardized β value**	***P* value**
**VFT, words**						
Model 1	−0.386	0.254^#^	−0.342	0.504	−0.869	0.015^*^
Model 2	−0.394	0.244^#^	−0.386	0.433	−0.873	0.016^*^
**CDT, score**						
Model 1	−1.063	0.464	−0.465	0.833	0.412	0.785
Model 2	−1.129	0.435	−0.688	0.745	0.419	0.784
**TMT-B, seconds**						
Model 1	0.042	0.585	0.277	0.021	0.335	0.000^#^
Model 2	0.019	0.809	0.224	0.057	0.342	0.000^#^

*Data are standardized β values, except for P value. Model 1 represents the relation between the index of spatial navigation performance and neuropsychological assessment data without adjustment; Model 2 represents the relation between the index of spatial navigation performance and neuropsychological assessment data adjusted for age, gender and education*.

* *P < 0.05*,

# *P < 0.01*.

## Discussion

In this work, the spatial navigation performance of SVD patients was shown to be poorer compared to healthy individuals, as reflected in “allocentric + egocentric” (for severe SVD patients only) and allocentric (for both severe and non-severe SVD patients) subtests, rather than egocentric subtest. Moreover, elderly patients with SVD exhibited cognitive deficits, such as mental flexibility, in comparison with healthy controls. Besides, the spatial navigation ability of elderly SVD patients during the allocentric subtest was associated with TMT-B and VFT performance. However, similar trends were not observed in these patients during “allocentric + egocentric” and egocentric subtests.

Spatial navigation is an essential human behavior that involves a multitude of cognitive processes interacting with one another (11). This kind of brand-new assessment is becoming more and more popular in clinical researches because it has fewer cultural, educational, and verbal biases compared to the existing cognitive tests (1). Different aspects of spatial navigation abnormalities have been reported in patients with neuropsychiatric disorders, including AD, persistent postural perceptual dizziness, traumatic brain injury, etc (5, 12, 13). Our results indicated that elderly patients with severe SVD exhibited poorer spatial navigation in both allocentric+egocentric subtest and allocentric subtest compared to healthy elderly subjects, indicating that severe SVD patients may encounter the problem of wayfinding in their daily life.

Although both allocentric and egocentric navigation strategies can be combined to attain optimum cognitive functioning, several studies (9, 14, 15) reported that a specific aspect of spatial navigation strategy dysfunction might imply the underlying pathophysiological processes. For example, autism spectrum disorder patients showed particularly difficulties in allocentric navigation, leaving egocentric navigation intact (14); LI patients performed worse than healthy control subjects during egocentric subtest (9); and AD patients exhibited significant both allocentric and egocentric navigation impairments relative to control individuals (15). In this study, elderly patients with SVD performed significantly worse on allocentric and “allocentric + egocentric” subtests, rather than egocentric subtests, when compared to healthy elderly subjects. This may be explained by the fact that disease burden disproportionately affects the network between the prefrontal cortex, hippocampus, and retrosplenial cortex (3, 16).

The relationship between spatial navigation and cognitive impairment has been explored recently. Laczo et al. (17) found that spatial navigation was not, or marginally, associated with most cognitive functions (attention, working memory, executive functions, verbal memory, and language learning) in mild cognitive impairment patients and healthy individuals. On the contrary, Brown et al. (18) proposed a significant association between spatial navigation, episodic memory, and executive functions. In the present study, we found a close relationship between allocentric spatial navigation and executive function/cognitive flexibility as well as semantic memory. However, similar trends were not observed for “allocentric + egocentric” or egocentric spatial navigation. The discrepancy between these studies could be explained by the distinct choice of cognitive assessment and distinct groups of individuals recruited. For example, Parizkova et al. (19) discovered that patients suffering from AD preferred more obviously egocentric strategies to allocentric strategies relative to healthy controls. Our findings illustrated that allocentric navigation strategy and executive function/cognitive flexibility might share the same functional brain area in aged SVD patients and the elderly.

Several limitations of this study need to be pointed out. First, the sample size was not large. Second, there is a lack of comprehensive assessment protocol, especially for Trail Making Test-A, as spatial navigation can potentially be affected by poor attention, cognitive flexibility, and psychomotor speed. Therefore, future research should encompass more study subjects and neuropsychological tests to address these shortcomings.

In summary, SVD patients showed spatial navigation deficits, especially on the allocentric navigation subtest. The allocentric navigation impairment was associated with TMT-B and VFT, rather than CDT performance, which are representations of cognitive flexibility and semantic memory, respectively. Furthermore, spatial navigation might serve as a promising tool to reflect a cognitive decline in elderly patients with SVD.

## Data Availability Statement

The raw data supporting the conclusions of this article will be made available by the authors, without undue reservation.

## Ethics Statement

The studies involving human participants were reviewed and approved by the ethics committee of the Seventh Medical Center of PLA General Hospital. The patients/participants provided their written informed consent to participate in this study.

## Author Contributions

YH and HT carried out the study concept and design. LC and HZ handled the acquisition of data. YZ carried out the analysis and interpretation of data. LC and HZ performed the drafting of the manuscript. All authors contributed to the article and approved the submitted version.

## Funding

This work was supported by the Wu Jieping Foundation (Grant No. 320.6750.18456) and Foundation CJ17J01.

## Conflict of Interest

The authors declare that the research was conducted in the absence of any commercial or financial relationships that could be construed as a potential conflict of interest.

## Publisher's Note

All claims expressed in this article are solely those of the authors and do not necessarily represent those of their affiliated organizations, or those of the publisher, the editors and the reviewers. Any product that may be evaluated in this article, or claim that may be made by its manufacturer, is not guaranteed or endorsed by the publisher.
