# Supplementary material for: Spatial Navigation Is Impaired in Elderly Patients With Cerebral Small Vessel Disease
Source: Front Neurol. 2021 Sep 8;12:608797. doi: 10.3389/fneur.2021.608797 (PMC8455869; doi:10.3389/fneur.2021.608797)
Supplement: Supplementary file 1 [file Data_Sheet_1.docx]

Supplementary Material 1

Fazekas scale to rate severity of white matter hyperintensities

A modification of suggested rating scales was used to describe the different types of hyperintense signal abnormalities surrounding the ventricles and in the deep white matter.

Periventricular hyperintensity (PVH) was graded as 0 = absence, 1 = "caps" or pencil-thin lining, 2 = smooth "halo," 3 = irregular PVH extending into the deep white matter.

Separate deep white matter hyperintense signals (DWMH) were rated as 0 = absence, 1 = punctate foci, 2 = beginning confluence of foci, 3 = large confluent areas.
